# P30 An unusual infective cause of inflammatory arthritis

**DOI:** 10.1093/rap/rkad070.051

**Published:** 2023-09-27

**Authors:** Shivani Gor, Verni Sopan, Simon Epps, Sarah Horton, Sarah Tansley

**Affiliations:** Royal National Hospital For Rheumatic Diseases, Bath, United Kingdom; Royal National Hospital For Rheumatic Diseases, Bath, United Kingdom; University Hospitals Bristol and Weston NHS Foundation Trust, Bristol, United Kingdom; University Hospitals Bristol and Weston NHS Foundation Trust, Bristol, United Kingdom; Royal National Hospital For Rheumatic Diseases, Bath, United Kingdom

## Abstract

**Introduction:**

Inflammatory arthritis can be a manifestation of a range of different diseases and the differential diagnosis at presentation is wide including a range of autoimmune, autoinflammatory and infective causes. Infective precipitants are important to identify as they are typically either self-limiting illnesses e.g. parvovirus B19 or can be treated with appropriate antibiotic therapy e.g. gonococcal arthritis. As autoimmune inflammatory arthritis, including seronegative disease is so common, a high degree of suspicion may be required to identify such cases. Here we present an unusual cause of infective inflammatory arthritis in a 46-year-old man.

**Case description:**

A 46-year-old male presented to the rheumatology clinic with inflammatory joint pain and swelling affecting his hands, elbows, knees and feet. An erythematous rash, sore throat and mouth ulcers had initially been present. The rash began on ankles and later spread to his torso. He described intermittent mouth ulcers and denied ulceration elsewhere.

Examination revealed injected conjunctivae and hypopigmented scaly macules on the skin with mild erythema. There was active synovitis at the wrists, PIPJs and elbows.

Blood tests showed a lymphocytic leucocytosis with elevated inflammatory markers and rheumatoid factor>100. ANA was weakly positive but ENA negative. X-rays of his hands and chest were normal. Infection screening included parvovirus, EBV, hepatitis, HIV, Lyme disease and leptospirosis were negative.

He was initially managed with a trial of prednisolone with the tapering regime. A month later he had persistent inflammatory joint symptoms which had responded to steroid therapy but recurred as steroids were weaned and so methotrexate therapy was initiated. This seemed to have a mild effect on his joint pains. However, as the prednisolone was weaned below 10mg his systemic symptoms of rash, fatigue and mouth ulcers recurred. He had also developed cervical lymphadenopathy. His prednisolone was increased again.

He presented to the local ophthalmology team with a deterioration in his vision and was diagnosed with cataracts. His vision deteriorated further and severe bilateral panuveitis was diagnosed which was unresponsive to prednisolone. He was referred on to a tertiary ophthalmology team. Signs of retinal arteritis and phlebitis raised the suspicion of infective rather than non-infective uveitis. In addition, there was optic atrophy consistent with previous optic neuritis. Further testing including a syphilis serology screen revealed active syphilis infection.

Appropriate antibiotic therapy was initiated through sexual health services. His methotrexate stopped and prednisolone weaned. He made a good response with the antibiotics.

**Discussion:**

Our patient demonstrates a case of secondary syphilis presenting with inflammatory arthritis and uveitis. He was initially managed as a seronegative inflammatory arthritis. Inflammatory arthritis can be seen in up to 10% of secondary syphilis cases. Some reports describe polyarthralgia and affected joints can include the knees, wrists, ankles and sacroiliac joints. Typically, inflammatory arthritis associated with syphilis manifests 3-12weeks into the secondary stage, but timings can vary. Patients do describe characteristic morning stiffness in the affected joints which does improve with movement. Commonly joint symptoms are accompanied by a non-pruritic, erythematous maculopapular rash, like what our patient experienced. However, this tends to involve the palms and soles.

It is unclear if syphilitic arthritis represents an immune phenomenon or occurs via inoculation of the joint. To date, there have been no case reports that describe spirochetes being isolated in a joint aspirate but one case published in France did report positive syphilis PCR in synovial fluid. The synovial fluid was described as thick, turbid and yellow.

The second key symptom that our patient suffered with was uveitis. His uveitis progressed despite high doses of prednisolone. Ocular manifestations of syphilis can affect any part of the eye but uveitis is the most common; optic neuritis, cranial neuropathies and pupil abnormalities may occur. It is seen in sexually active patients of all age groups and any sex. Syphilis should be considered for all patients with uveitis regardless of the anatomical location and phenotype of uveitis although there are some phenotypes well-recognised to occur in syphilitic infection.

Infectious tenosynovitis has also been reported and this occurs via haematogenous spread of T. pallidum. Reports describe involvement of the tenosynovium which can present as joint swelling and dactylitis. If the infection extends beyond the tenosynovium to the skin and nails it can lead to onycholysis.

**Key learning points:**

Syphilis is a sexually transmitted disease caused by the bacterium Treponema pallidum. Diagnoses in the UK increased 15% in 2022, compared to 2021 and 8.1% compared to 2019. Syphillis incidence in the UK is currently the highest level since 1948. Syphilis is a multisystem disease which can involve the skin, cardiovascular and neurological systems. Arthritis is not a common manifestation, but it is seen in the congenital form and it can also be a rare sequalae of acquired syphilis. Our case reports highlights the importance of including syphilis in the differential diagnosis of patients presenting with a variety of musculoskeletal symptoms. In the case presented, an underlying infectious cause was considered early, but VDRL testing not included in the initial ‘infection screen’.

Syphillis incidence in the UK is currently the highest level since 1948. As the prevalence of syphilis continues to rise, healthcare professionals should have a low threshold to screen for syphilis.

People aged 15 to 24 years remain the most likely to be diagnosed with sexually transmitted infections. Testing for syphilis should be considered in any patient presenting with multisystem disease, particularly rash, uveitis, ulceration and systemic upset. Moreover, if a patient is not improving despite adequate immunosuppression and continues to have multisystem symptoms, it is important to re-test patients.

Screening for syphilis prior to immunosuppression may be necessary for high-risk patients, especially with the rising use of biologic therapies. There have been cases of reactivation of syphilis on initiation of treatment of rheumatology conditions with anti-TNF and anti-IL17 medications.

Rheumatologists are familiar with multisystem diseases including sarcoidosis and lupus known to be great mimickers and heterogeneous in their presentation. Particularly given the increasing incidence, we suggest syphilis is added to the differential diagnosis and screened for routinely when infectious causes are being considered.

